# Successful conservative management of a spontaneous intraperitoneal urinary bladder rupture secondary to benign prostatic hyperplasia: a case report

**DOI:** 10.11604/pamj.2021.38.268.26684

**Published:** 2021-03-16

**Authors:** Ahmed Ibrahimi, Hamza Dergamoun, Idriss Ziani, Zayd El Boukili El Makhoukhi, Hachem El Sayegh, Lounis Benslimane, Yassine Nouini

**Affiliations:** 1 Department of Urology A, Ibn Sina University Hospital, Faculty of Medicine and Pharmacy, Mohammed V University, Rabat, Morocco

**Keywords:** Conservative treatment, intraperitoneal bladder rupture, acute urinary retention, urinary ascites, case report

## Abstract

A spontaneous intraperitoneal bladder rupture is a rare, serious and life-threatening surgical emergency of various etiologies, with unspecific clinical presentation, and difficult diagnosis. Surgical treatment is the standard therapy for intraperitoneal bladder rupture; however, there is an increasing tendency toward conservative management in selected patients with favorable characteristics. Herein, we report a rare case of a 65-year-old male patient presented to the emergency department with intraperitoneal bladder rupture following an episode of acute urinary retention due to benign prostatic hyperplasia, and which was successfully managed conservatively with urinary bladder catheterization and antibiotic therapy, without any complication.

## Introduction

A spontaneous intraperitoneal bladder rupture is an extremely rare urologic emergency, potentially life-threatening with significant morbidity and mortality rate reaching up to 50%, and mostly occurs in men [1]. Etiologies are dominated by underlying bladder diseases, bladder outflow obstruction, and iatrogenic causes. Clinical presentation is non-specific, and the diagnosis should be suspected in the presence of signs of acute peritonitis associated to lower urinary tract symptoms [2]. Despite that there are still no published guidelines for therapeutic management, surgical repair remains the standard treatment for intraperitoneal bladder rupture. However, there is an increasing tendency toward conservative management in selected patients, with bladder catheterization, systemic antibiotics associated in some cases with percutaneous peritoneal drainage [2, 3]. Herein, we report a rare case of spontaneous intraperitoneal bladder rupture in a 65-year-old patient, secondary to acute urinary retention, due to benign prostatic hyperplasia, and which was successfully managed conservatively without any complication.

## Patient and observation

**Figure 1:**
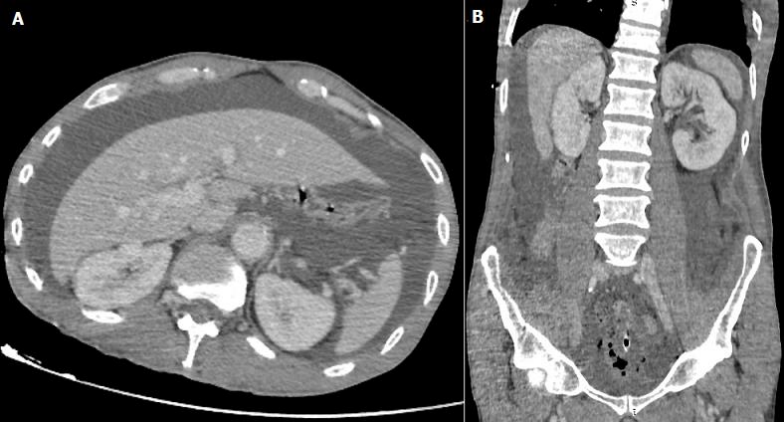
A) axial view on CT scan showing large amounts of intraperitoneal fluid; B) coronal reconstruction of the CT scan showing large amounts of ascites, associated with free intraperitoneal air in the pelvis

**Figure 2:**
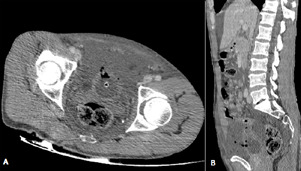
CT abdomen and pelvis revealing large amounts of free intraperitoneal fluid, leaking from the bladder through a small defect in the anterior bladder wall suggesting perforation, associated to intraperitoneal pelvic free air, with infiltrated perivesical fat, and empty bladder; A) axial view; B) sagittal reconstruction

**Figure 3:**
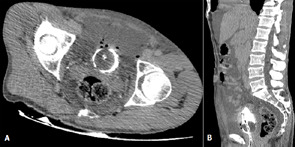
late excretory phase showed no contrast extravasation into the peritoneal cavity, and a small defect in the anterior bladder wall sealed with inflated urinary catheter balloon, with large amounts of free intraperitoneal fluid and pelvic free air; A) axial view; B) sagittal reconstruction

A 65-year-old male patient, with a past medical history of benign prostatic hyperplasia (BPH) not followed for 3 years, presented to the emergency department with generalized malaise, painful abdominal distension, and absence of urine outflow, following an episode of acute urinary retention for 1 day. On examination, he was apyretic, his abdomen was distended and painful, especially in the hypogastric area, without guarding or rebound tenderness, with empty bladder, and absence of urine outflow. Digital rectal examination revealed an enlarged prostate, with firm consistency with no palpable nodule. Vital parameters were normal, with blood pressure of 123/72 mmHg, heart rate of 78 beats/min, and respiratory rate of 18 breaths/min. Urinary bladder catheterization allowed to drain approximately 60 ml of clear urine. Computed tomography (CT)-urography revealed large amounts of intraperitoneal fluid, leaking from the bladder through a small defect in the anterior bladder wall, associated to free intraperitoneal air, with an infiltrated perivesical fat, and empty bladder (Figure 1, Figure 2), late excretory phase showed no contrast extravasation into the peritoneal cavity, because the bladder defect was sealed with inflated urinary catheter balloon (Figure 3). The diagnosis of intraperitoneal bladder rupture secondary to acute urinary retention due to benign prostatic hyperplasia was subsequently retained.

Given the patient's stable clinical status, early diagnosis, the absence of sepsis or associated comorbidities, conservative management has been decided, it consisted of intravenous antibiotic with close observation and maintenance of bladder catheterization. Abdomen distension has slowly abated over the next day. Laboratory testing revealed hemoglobin of 12.7g/dL, with normal serum creatinine and urea level, negative C-reactive protein (CRP), and negative urine culture. On the seventh day, cystogram revealed no evidence of any contrast leak, the cystoscopy did not show any suspicious bladder lesions, with enlarged obstructive prostate. The patient was discharged from the hospital at the 8^th^ day in good health with indwelling bladder catheter, and a transurethral resection of the prostate (TURP) was planned after 4 weeks.

## Discussion

A spontaneous urinary bladder rupture is an extremely rare pathological entity, with an incidence of 1: 126,000, and a mortality rate of around 50% [1, 4]. Predisposing conditions are associated with weakening of the bladder wall and/or increase in intra-vesical pressure. The most common causes are malignancy, pervious pelvic radiotherapy, chronic bladder infection or inflammation, neurogenic bladder, alcohol intoxication, bladder diverticulum, and bladder outflow obstruction [1, 2]. In our case bladder rupture seems to be the results of increase in intra-vesical pressure secondary to sudden bladder distension and a decrease of the strength of the bladder wall due to chronic prostate obstruction.

Clinical presentation is non-specific and vague, and the diagnosis has been described as challenging in most of cases, it manifests by haematuria, abdominal distension, diffuse tenderness in abdomen, associated with decreased urine output and voiding complaints, or sometimes it takes the clinical picture of acute peritonitis with sepsis, oliguria and acute renal failure due to reabsorption of urine through the peritoneum, especially when the diagnosis is delayed or missed [2, 4]. This atypical presentation can delay diagnosis and treatment and lead to severe complications, and can be confused with other causes of acute peritonitis, and in some cases the diagnosis is only made intraoperatively [1].

CT-cystography is the investigation of choice for a suspected urinary bladder rupture, with sensitivity and specificity of 78% and 99%, respectively, it offers the advantage of exploring the whole abdominal cavity and etiological research in the same examination. CT-urography with excretory phase can also make diagnosis, it is less invasive than CT-cystography and provides a complete overview of the entire excretory system, but it can sometimes miss the diagnosis [5].

Once urinary bladder rupture is diagnosed, treatment depends on type of bladder perforation as well as the nature of an underlying pathology which can require additional treatment, this treatment is not codified and there are still no clear guidelines in the available literature [3]. Several authors advocate for surgical approach with immediate repair for intraperitoneal bladder rupture, with peritoneal drainage, which can be made by laparotomy or laparoscopy [2, 3]. Nonoperative management can be successfully considered in selected patients with favourable characteristics, it consists of urinary bladder catheterization associated to antibiotics and percutaneous peritoneal-fluid drainage in case of incomplete initial drainage by urethral catheter alone, with close monitoring and surgical intervention at the slightest doubt [2, 4]. In our patient and given its favourable characteristics, nonoperative management proved successful without any complication.

These findings highlight that bladder rupture can be a serious complication of benign prostatic hyperplasia, which is the most common pathology in elderly man, and must be treated effectively before the occurrence of irreversible complications. Conservative management may be a valid treatment option for patients diagnosed with a spontaneous intraperitoneal bladder rupture. However, further research is needed to determine the best candidates for such approach.

## Conclusion

Spontaneous intraperitoneal bladder rupture is a rare urological emergency. Prompt and accurate diagnosis is the key for appropriate management. Non-operative treatment with bladder drainage, and antibiotic therapy could be a valid option in carefully selected patients.
